# Seasonal Habitat Use by Greater Sage-Grouse (*Centrocercus urophasianus*) on a Landscape with Low Density Oil and Gas Development

**DOI:** 10.1371/journal.pone.0165399

**Published:** 2016-10-27

**Authors:** Mindy B. Rice, Liza G. Rossi, Anthony D. Apa

**Affiliations:** 1 Colorado Parks and Wildlife, Avian Research, Fort Collins, Colorado, United States of America; 2 Colorado Parks and Wildlife, Terrestrial Section, Steamboat Springs, Colorado, United States of America; 3 Colorado Parks and Wildlife, Avian Research, Grand Junction, Colorado, United States of America; Universidade de Lisboa Instituto Superior de Agronomia, PORTUGAL

## Abstract

Fragmentation of the sagebrush (*Artemisia spp*.*)* ecosystem has led to concern about a variety of sagebrush obligates including the greater sage-grouse (*Centrocercus urophasianus*). Given the increase of energy development within greater sage-grouse habitats, mapping seasonal habitats in pre-development populations is critical. The North Park population in Colorado is one of the largest and most stable in the state and provides a unique case study for investigating resource selection at a relatively low level of energy development compared to other populations both within and outside the state. We used locations from 117 radio-marked female greater sage-grouse in North Park, Colorado to develop seasonal resource selection models. We then added energy development variables to the base models at both a landscape and local scale to determine if energy variables improved the fit of the seasonal models. The base models for breeding and winter resource selection predicted greater use in large expanses of sagebrush whereas the base summer model predicted greater use along the edge of riparian areas. Energy development variables did not improve the winter or the summer models at either scale of analysis, but distance to oil/gas roads slightly improved model fit at both scales in the breeding season, albeit in opposite ways. At the landscape scale, greater sage-grouse were closer to oil/gas roads whereas they were further from oil/gas roads at the local scale during the breeding season. Although we found limited effects from low level energy development in the breeding season, the scale of analysis can influence the interpretation of effects. The lack of strong effects from energy development may be indicative that energy development at current levels are not impacting greater sage-grouse in North Park. Our baseline seasonal resource selection maps can be used for conservation to help identify ways of minimizing the effects of energy development.

## Introduction

Fragmentation from energy development may result in changes in land cover and could alter the spatial patterns of a species’ habitat use [1]. The ability to identify priority habitat, particularly for species of conservation concern, is an important and urgent management concern [2]. The greater sage-grouse (*Centrocercus urophasianus*) (GRSG) is a species of conservation concern due to population declines and range contraction and is dependent on the sagebrush ecosystem [3]. In 2015, the Fish and Wildlife Service (USFWS) determined that GRSG were not warranted for protections as a threatened or endangered species, but a status review will be conducted in five years [4]. GRSG habitat associations have been well documented at multiple scales and are strongly tied to sagebrush landscapes although utilization of those landscapes can differ depending on the season and the bird’s life stage [5]. In general, seasonal habitats for GRSG are based on life stages including breeding (including lekking and nesting), summer (including late brood-rearing), and winter [5]. Seasonal habitat use is an important consideration when developing models to predict habitat use as GRSG use distinct seasonal habitats throughout their annual cycle [2]. Developing these seasonal habitat maps for GRSG is a necessary component of managing human disturbance [6], but even more important in areas where development has not yet begun.

Loss and degradation of native vegetation has affected much of the sagebrush ecosystem in Western North America and it has become increasingly fragmented [2]. The sagebrush ecosystem only occupies about 56% of its historic range and is being degraded and fragmented by multiple factors including anthropogenic development [7]. One such anthropogenic development is the infrastructure associated with energy extraction [7]. Infrastructure associated with energy development including pipelines, roads, and well pads not only directly impacts native sagebrush, but also can serve as a vector for the introduction of invasive species which furthers fragmentation [8]. In the United States, domestic energy production is encouraged to reduce dependence on foreign energy sources and much of this development will occur in sagebrush and grassland habitat [9].

Energy development has emerged as a major issue in GRSG conservation because areas currently under development for energy contain some of the highest densities of GRSG [10]. The number of producing wells within the range of GRSG has tripled from the 1980s to 2007 and the impacts at conventional well densities (8 well pads per 2.6 km^2^ on public lands) are exceeding the species’ threshold of tolerance [10]. Previous research examining the effects of energy development on habitat use suggests that GRSG populations are negatively affected by energy development activities, especially those that degrade important sagebrush habitat [10–12]. This often results in a decrease in available habitat or the avoidance of critical seasonal habitat [13–15]. Most of these studies have been conducted in areas where energy development already exists at high levels of development.

**Fig 1:**
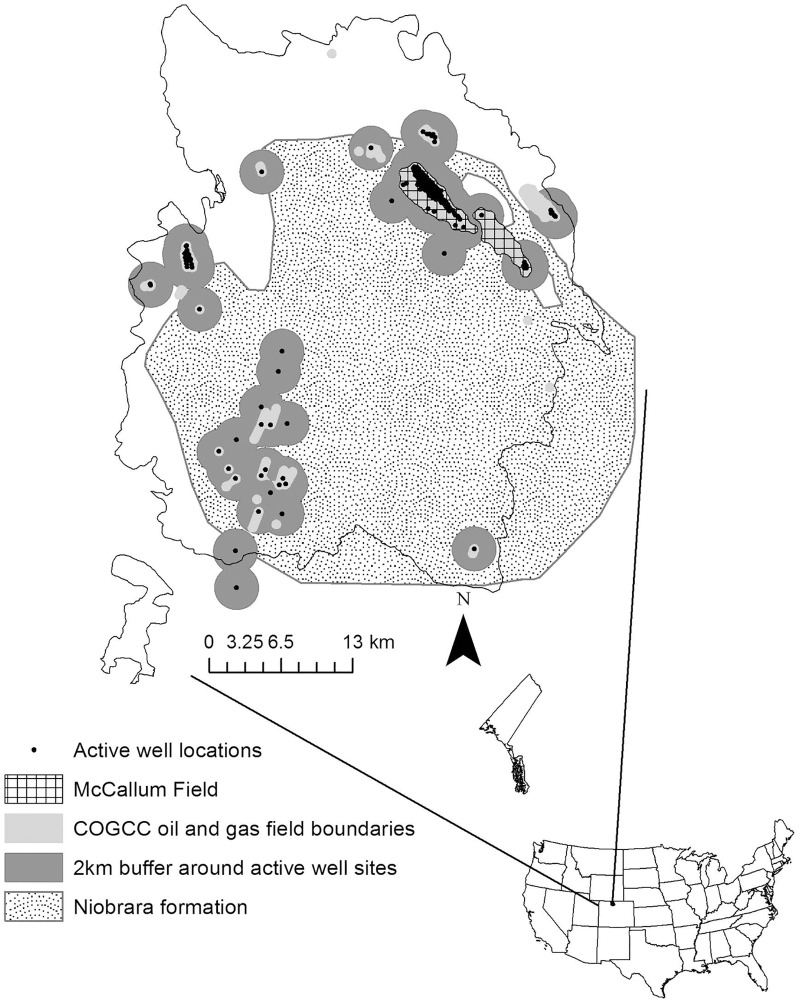
Study Area. The North Park study area in Northwest Colorado, USA along with the associated energy development during our study period (2010–2012). Energy development includes active well locations, oil and gas fields, 2 km boundaries used for local scale development models, McCallum field, and the Niobrara formation.

North Park is 1 of 6 distinct populations of GRSG in Northwestern Colorado (Fig 1) and is considered one of the largest and most stable consisting of approximately 20% of the statewide population [16]. North Park is subject to a relatively low level of energy development compared to other populations both within and outside the state. There was an estimated 4.1% of the 1,606 km^2^ land surface for this population directly classified as an oil/gas field (Colorado Oil and Gas Conservation Commission (COGCC) [17]) during the period of our study. The highest well pad density in North Park is located within the McCallum Field [18], which constitutes < 2% of the surface area and 66% of the wells in North Park, with an estimate of 3.9 wells per km^2^ during our study period (119 wells in 30.4 km^2^). In all of North Park the density is much lower at 0.11 wells per km^2^ during our study period (181 wells in 1,606 km^2^) and without including McCallum field, the estimate is reduced to 0.04 wells per km^2^. In contrast, other GRSG populations in Colorado and Wyoming have more extensive development with well densities ranging from 0.19–1.58 wells/km^2^ (Table 1). Most of these studies indicated negative relationships between energy development variables and resource selection for GRSG (Table 1). Although the current energy development situation in North Park is mostly low and concentrated in a few oil/gas fields, 99% of North Park is considered high for potentially valuable oil/gas resources [17] indicating a huge potential for development. Given the relatively low level of existing energy development in North Park and the potential for more oil/gas leases, current seasonal habitat selection could provide a useful baseline with which to examine the effects future energy development may have on GRSG distribution and habitat use [10]. North Park provides a unique case study to look at seasonal habitat selection of a healthy GRSG population in an area with low level energy development prior to what could be expanded energy extraction.

**Table 1:** GRSG resource selection studies and their response to oil/gas wells.

Study	Season	State	Density of wells per km^2^ in study area	Overall response to oil/gas wells
Walker et al. 2016[19]	All Seasons	Colorado	0.28 (365 wells in 1290 km^2^)	NA
Fedy et al. 2015[2]	Breeding	Wyoming	1.58 (2548 wells in 1612 km^2^)	negative (pre-mitigation)
Kirol et al. 2015[1]	All Seasons	Wyoming	0.54 (600 wells in1093 km^2^)	negative
Smith et al. 2014[15]	Winter	Colorado/Wyoming	0.41 (2512 wells in 6093 km^2^)	negative
Dzialak et al. 2012[20]	Winter	Wyoming	0.19 (1052 wells in 5625 km^2^)	negative
Doherty et al. 2008 [14]	Winter	Wyoming/Montana	1.21 (29,000 wells in 24,000 km^2^)	negative
Walker et al. 2007 [11]	Breeding	Wyoming/Montana	1.12 (28,000 wells in 24,000 km^2^)	negative

Resource selection studies for greater sage-grouse that evaluated responses to anthropogenic disturbance due to oil/gas wells including the season of use, the state of the study area location, density of wells in the study area, and overall pattern of resource selection to oil/gas wells. The overall pattern indicates whether GRSG had a positive, neutral, or negative impact due to oil/gas wells.

We had two main objectives for this study. First, we developed baseline seasonal habitat selection models using GRSG radio-telemetry data collected in the North Park population. We predicted that sagebrush (*Artemisia spp*) would be an important factor for habitat selection during the breeding, summer, and winter seasons and most of North Park would be suitable habitat. Second, we used energy development variables to evaluate the degree, if any, to which low levels of energy development in North Park currently influences GRSG habitat selection at two scales. We predicted that GRSG would avoid energy development particularly given that there is abundant habitat in the region far from oil and gas infrastructure and activities.

## Materials and Methods

### Study area

Our study area was located in Jackson County, Northwestern Colorado (Fig 1). Jackson County is bounded on the north by Wyoming, the west by the Park Range, the south by the Rabbit Ears Range, and the east by the Medicine Bow Range. This semi-arid basin is commonly referred to as North Park. GRSG occupied range in North Park encompasses approximately 1,606 km^2^ and includes the majority of the North Park basin. The North Park basin is largely comprised of a mix of big sagebrush (*A*. *tridentata*) uplands interspersed with riparian and wetland communities and irrigated agriculture located along multiple waterways throughout North Park that are the headwaters for the North Platte River. The human population in Jackson County is considered rural with less than one person per 2.59 km^2^ and a total population of 1,392 reported in 2010 Census [21].

### Data

GRSG were captured and radio-marked during April 2010 and April 2011 using spot-lighting techniques [22–23] and ATVs. Following release, the movements and survival of all radio-marked GRSG were monitored approximately once per week until February 2012. We collected telemetry locations from 117 female GRSG over the study period for this analysis. All GRSG captured were weighed (±1 g) using an electronic scale and marked with uniquely numbered aluminum leg bands. We fitted females with 17-g VHF necklace-mounted radio transmitters (Model A4330; Advanced Telemetry Systems, Inc., Insanti, MN) with a 30 cm antenna lying between the wings and down the back of the grouse. Transmitters had a minimum battery life of 18 months and a 4-hour mortality signal. The radio transmitter package was 1.0% and 1.2% of the body weight for adult and yearling females, respectively. Trapping and handling protocols were approved by the Colorado Parks and Wildlife Animal Care and Use Committee (Permit # 01–2010). The study species was not federally protected during the period of our study. Our GRSG research was conducted on a combination of public (U. S. Fish and Wildlife Arapahoe National Wildlife Refuge and Bureau of Land Mangement) and private land. We gained permissions for access to property from the appropriate landowner.

Incubating females were monitored more frequently (>1 time per week) to determine nest fate. Each time a grouse was located, it was circled at a 50–100 m radius to determine habitat type while at the same time avoiding flushing the bird. This radius could be larger during the winter months when birds were grouped in flocks and flushed more easily. A precise Universal Transverse Mercator (UTM) location was not possible at the time of location (the birds were not intentionally flushed). To obtain more precise locations, the observer marked a waypoint approximately 50 m in one of the 4 cardinal directions from the estimated location of the bird then manually corrected the location. At each location, date, time, UTM coordinates, slope and aspect were recorded. A fixed-wing aircraft assisted to locate any grouse not located from the ground or lost during ground monitoring efforts.

Locations were assigned to one of three seasons: breeding (1 April– 15 July), summer (16 July– 1 September), and winter (1 October–1 March). We estimated the average daily movement distances across all the birds in each season by dividing the average distance between locations by the average days between locations. We used these distances to buffer all presence locations within that season (150.8 m in the breeding season, 203.6m in the winter season, and 83.1m in the summer season). We used this buffer to account for possible error in the telemetry locations as well as to summarize the environmental covariates at a biologically relevant scale. We randomly generated a sample of “available” locations within the same geographical extent of the North Park basin [24]. We conducted a sensitivity analysis of the available sample size for each season [25]. To do this, we generated a resource selection function (RSF) for an availability sample of N = 1,000, 2,000,…10,000. We found that RSF coefficients converged at N = 7,000 available samples in the breeding and winter seasons and at N = 6,000 in the summer season. We applied the same daily movement buffer specific to each season to these available locations. We summarized all habitat variables within these buffers.

We classified vegetation type into 15 biologically meaningful categories (S1 Appendix) using the basinwide vegetation layer (i.e. sagebrush, forest, etc). This land cover layer was constructed from 25-m resolution landsat imagery as part of the Colorado Vegetation Classification Project administered by the former Colorado Division of Wildlife (currently Colorado Parks and Wildlife) in collaboration with the BLM and the U.S. Forest Service and was completed in 2005. Because North Park is sparsely populated, has low anthropogenic disturbance, and no relevant environmental perturbations (e.g. wild or managed fire), we believe the vegetation cover types still reflect current conditions in this area. From those 15 categories, we excluded those that consisted of < 0.1% of the land surface within the buffers (e.g. residential, bare, greasewood/herbaceous (*Sarcobatus vermiculatus*), bitterbrush (*Purshia tridentata*), talus, alpine, and water). In addition, we removed aspen (*Populus tremuloides*) and forest because models did not converge due to the lack of these variables in the presence buffers in all seasons. Thus, the vegetation categories we used in model development were irrigated agriculture, sagebrush, grassland, sagebrush/grassland, and riparian, which have all been shown to be important predictors of GRSG habitat in previous studies (S1 Appendix; [15, 19, 26–28]). We also updated the irrigated agriculture vegetation class from the basinwide layer using the Colorado Division of Water Resources District 47 irrigated agriculture shapefile from 2011 [29]. We did this to accurately reflect the locations of irrigated agriculture during the specific time period of our study. The proportion of each of these vegetation categories were summarized within the seasonal buffers for use in the models.

We obtained elevation data from the U.S. Geological Survey (USGS) digital elevation model and used the national hydrography dataset to measure the average distance to perennial water sources as well as the density of water within each buffer [20]. We also included the Normalized Difference Vegetation Index (NDVI), a measure of plant primary productivity which has been used in recent GRSG studies [1, 26, 30]. A plant that is actively photosynthesizing will absorb most of the visible light resulting in a higher NDVI value and higher plant productivity [31]. We also measured the distance to sagebrush for those locations that did not contain sagebrush within their buffers. Particularly in the summer, GRSG use other habitat types within the sagebrush complex [5, 20, 32–34] and Dunn and Braun [35] found that managers might use 150–200 m as a guideline for the interspersion of cover types on GRSG summer range. Dunn and Braun [35] reported observations of GRSG feeding on the edges of vegetation cover that was higher in vertical and horizontal structure. In addition, Klott and Lindzy [36] reported that GRSG avoided large openings in meadows while feeding near the ecotone with sagebrush. Although most of our presence locations included sagebrush within their buffers, it was important to know the distance to sagebrush from those locations without a direct sagebrush component. We also included a distance to irrigated agriculture as there are large riparian corridors with irrigated agriculture in North Park that dissect the study area. The distance to agriculture and the distance to sagebrush variables were included as a way to measure possible edge effects between habitats in North Park (Table 2).

**Table 2:** Habitat variables used to model greater sage-grouse selection.

Variable	Breeding	Summer	Winter	Available
Sagebrush^a^	0.80	0.47	0.78	0.50
Grassland^a^	0.03	0.09	0.03	0.07
Sagebrush/grassland^a^	0.11	0.13	0.10	0.10
Riparian^a^	0.01	0.02	0.00	0.01
Irrigated agriculture^a^	0.04	0.24	0.04	0.21
Elevation	8251.1	8200.9	8204.7	8320.6
NDVI^b^	0.28	0.45	0.01	0.33
Water density (km/km^2^)	1.90	2.64	2.27	2.08
Distance to water (km)	0.25	0.15	0.20	0.24
Distance to sagebrush (km)^c^	0.01	0.05	0.01	0.09
Distance to agriculture (km)^c^	0.95	0.37	0.89	0.59

Variables used in greater sage-grouse habitat models with the mean value for presence and available buffers in the breeding (1 April- 15 July), summer (16 July– 1 September), winter (1 October– 1 March) seasons, and available buffers in North Park, Colorado, U.S.A (2010–2012).

^a^proportion of area covered by vegetation type

^b^Normalized Difference Vegetation Index

^c^if vegetation located within buffer, then distance is equal to “0”.

### Model building by season

We first calculated Pearson Correlation Coefficients (*r*) for all the habitat variables. In all three seasons, irrigated agriculture and NDVI were highly correlated with sagebrush (*r*>0.65) and we removed them from further model development and consideration. We also removed distance to sagebrush from the breeding and winter models and elevation was removed from the summer model, as these variables were correlated with sagebrush. We used a binomial generalized linear model with a logit link using the lme4 package in program R [37]. We used a random intercept for each individual grouse within each season to account for unbalanced sampling among animals [38].

We constructed a set of alternative models from all linear combinations of the habitat variables in each season [39]. We generated predictions from each of the models with weights that sum to 95% and averaged the predictions into a final model for each season to strengthen inference when there was not a clear combination of variables to construct a set of competing models (S2 Appendix, [40]). We reported the top model for each seasonal model as we did not use averaged coefficients, but rather averaged predictions. The coefficients for each of the seasonal models are equivalent to selection ratios [41] and exp(β_i_) can be interpreted directly as the odds ratios. Additionally, we calculated standardized coefficients to assess the relative effects of different covariates measured at different scales [42]. To do this we centered and scaled all variables in each season (mean = 0, SD = 1) [39].

We re-calculated the weights from the models in the 95% set to sum to 1 and we used each model within the set to create a model prediction surface [43]. We then multiplied each prediction surface within the model set by its weight and then added these models together to produce a final model averaged prediction surface [44]. This process allows all plausible models in a set to be used in multimodel inferences for spatial predictions [43].

We used the final model averaged prediction surface for each season to create a prediction surface in ArcMap 10.1 based on the associated habitat variables (ArcGIS 10.1; Environmental Research Systems Institute, Redlands, CA). We used the logistic function to create a surface proportional to probability of presence (hereafter relative probability) with values between 0 and 1 across the study area for each season (1 = high, 0 = low).

### Energy development impact analysis

Since energy development occurs in isolated patches in North Park, we approached the energy development analysis using two scales of analysis. First, we evaluated the impact for each season across all of North Park using the same presence and available buffers used in the base models (hereafter landscape scale). Second, since oil/gas wells in North Park were concentrated in smaller areas within our North Park study area and in an effort to not diffuse the influence of energy development across North Park, we conducted a second analysis at a smaller scale (hereafter local scale). This analysis restricted our dataset to only those GRSG presence and available buffers located near the oil/gas fields ([17]; Fig 1).

To delineate energy development, we obtained a shapefile from the COGCC website which outlined locations for wells, ownership, and well ID information [17]. Next, we determined historical dates for all 677 wells from the COGCC website and incorporated well status, spud date, completion date, first production date, last production date, and expiration date. We only included wells that were active (n = 181) during our study period and where the well pad could be seen visually on satellite and/or NAIP imagery [6].

Due to the lack of digital transportation data in Jackson County, Colorado, we created a database of historical road networks versus roads created for energy development purposes. We obtained shapefiles with existing highways, major county roads, and local roads [45]. We digitized roads utilized for energy development with the assistance of hard copy maps and digital topographic maps (Google Earth 6; Google Inc., Mountain View, CA). We used well pad coordinates and identified those roads leading towards oil/gas wells or those within an oil/gas field and classified these as roads created for the purpose of energy production (unless they were already classified as a county/local road by Colorado Department of Transportation).

We were also interested in the overall energy development intensity within the energy development areas and created an energy development index by combining both the density of oil/gas roads leading to active wells and the density of active wells within 1 km^2^. We first created an active oil/gas well pad density layer in ArcMap followed by an oil/gas road density layer. By combining these two layers, we created an index of energy development which was used to assess the effect of energy development within each season. We also included distance to oil/gas roads and distance to active well pads to evaluate distance effects in the models.

For the landscape scale analysis, we added energy development variables (distance to oil/gas roads, distance to active well pads, and energy development index) to the best base model for each season to evaluate if energy development variables were informative predictors of GRSG habitat use in North Park based on a reduced AIC_c_ [14]. We re-evaluated any correlations and found distance to oil/gas roads and distance to well pads were correlated for all seasons, so we removed distance to well pads. We used the base seasonal models as a starting point because we were interested in the influence of energy development variables on our “best” prediction surface. If the seasonal model fit improved with the addition of energy development variables, we applied the average predicted model to the raster surfaces to evaluate changes in the prediction surface.

For the local scale analysis, we delineated a focus area based on energy development analysis using oil/gas fields identified by the COGCC and then we applied a 2 km buffer around active wells [17]. We selected this distance because Remington and Braun [46] found that coal mining and oil field development in North Park resulted in decreased lek attendance on leks within 2 km of development activities. Although this information is dated, we feel that local information is superior and more informative than more general buffers [47]. We then selected within each season those presence and available buffers that overlapped the 2 km energy development buffer. We then followed the same procedure for adding energy variables as in the landscape scale analysis. At the local scale there was a correlation between distance to active wells and the energy development index so distance to wells was removed.

### Model validation

Common methods of evaluating model performance including Kappa and receiver operating curves are inappropriate for presence/available data as the distribution of used sites is drawn directly from the distribution of available sites [48]. Therefore, we performed a k-fold cross validation 5 times withholding 20% of data randomly for each iteration in program R [48–49]. We placed all locations for individual birds in either the model building set or the training set (i.e. locations for a single bird could not be in both the model building and training set). We assessed model evaluation for presences only as available locations were randomly chosen and not true absences [50]. For each data fold, the withheld data can be assessed against the model predictions of the training data using correlations between bin rank of the RSF values and the frequency of independent, withheld observations in the same bin rank standardized for area [51]. We assessed the relationship between predicted occurrence for withheld GRSG locations and their frequency within 10 incrementally higher relative probability of use bins of equal size adjusted for area [52–53].

## Results

### Bird capture and movements

We collected a total of 3,985 locations from GRSG in North Park from 95 female GRSG radio-marked during 2010 and 22 additional females in 2011. There were a total of 1,480, 874, and 1,631 breeding, summer, and winter locations, respectively (S1 Dataset). On average our movement buffers based on bird movement were 150.8 m, 203.6 m, and 83.1 m in the breeding, winter, and summer seasons and we were able to collect a telemetry location on each bird every 12.6 days, respectively. Our movement buffers for GRSG in North Park were larger during the winter season and smaller during the summer season. We found the only habitat variable common among all 3 seasons was distance to sagebrush, which was closer to presence locations than available locations (Table 2). We found that the summer season exhibited the most differences from the breeding and winter seasons based on mean values including a lower proportion of sagebrush and a higher proportion of grass, riparian, and irrigated agriculture in the presence buffers and the presence buffers being closer to irrigated agriculture (Table 2).

### Seasonal models

**Fig 2:**
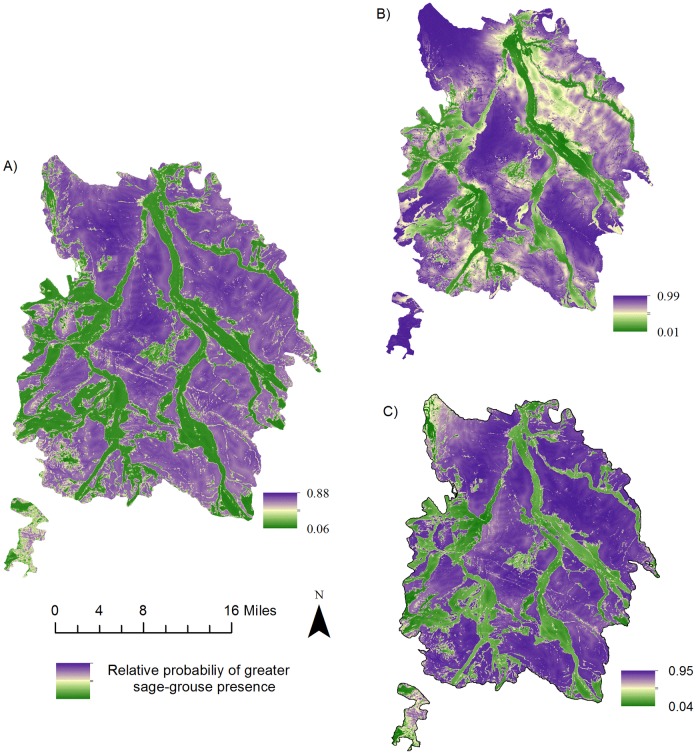
Relative probability of presence during the breeding season. Relative probability of female greater sage-grouse presence in North Park, U.S.A. (2010–2011) during the (A) breeding season (1 April– 15 July), (B) breeding season with the addition of the distance to oil/gas roads variable at a local scale, and (C) breeding season with the addition of the distance to oil/gas roads variable at the landscape scale.

Our model-averaged breeding season model included elevation, grassland, sagebrush, sagebrush/grassland, distance to irrigated agriculture, and distance to water (Table 3). The most important predictor was a positive relationship with sagebrush. We found the largest effects in the breeding season model came from strong positive relationships with sagebrush, grassland, and sagebrush/grassland vegetation and being further from irrigated agriculture (Table 3). Birds also showed a slight preference for lower elevations and closer to water (Table 3). The averaged prediction surface indicated large continuous landscapes of high quality sagebrush habitat in non-riparian areas (Fig 2A).

**Table 3:** Coefficients for the top model for the breeding season.

	STANDARDIZED COEFFICIENTS				UNSTANDARDIZED COEFFICIENTS				
Variable	β	SE	LCI	UCI	β	SE	LCI	UCI	Odds Ratio
Intercept	- 2.183				- 0.400				
Sagebrush (proportion)	1.283	0.071	1.180	1.386	3.161	0.176	2.908	3.413	23.585
Grassland (proportion)	0.162	0.054	0.085	0.240	1.456	0.481	0.764	2.148	4.289
sagebrush/grassland (proportion)	0.427	0.039	0.371	0.483	2.775	0.252	2.412	3.138	16.039
Elevation (ft)	- 0.118	0.062	- 0.208	- 0.029	- 0.001	0.0002	- 0.001	0.000	0.999
distance to agriculture (km)	0.124	0.037	0.071	0.177	0.167	0.049	0.096	0.238	1.181
distance to water (km)	- 0.094	0.038	- 0.148	- 0.040	- 0.435	0.174	- 0.686	- 0.184	0.647

Top model standardized and unstandardized coefficients for greater sage-grouse resource selection in North Park, Colorado, U.S.A. (2010–2011) in the breeding season (1 April- 15 July), including the standard error (SE), lower (LCI) and upper (UCI) 95% confidence intervals, and odds ratios.

**Fig 3:**
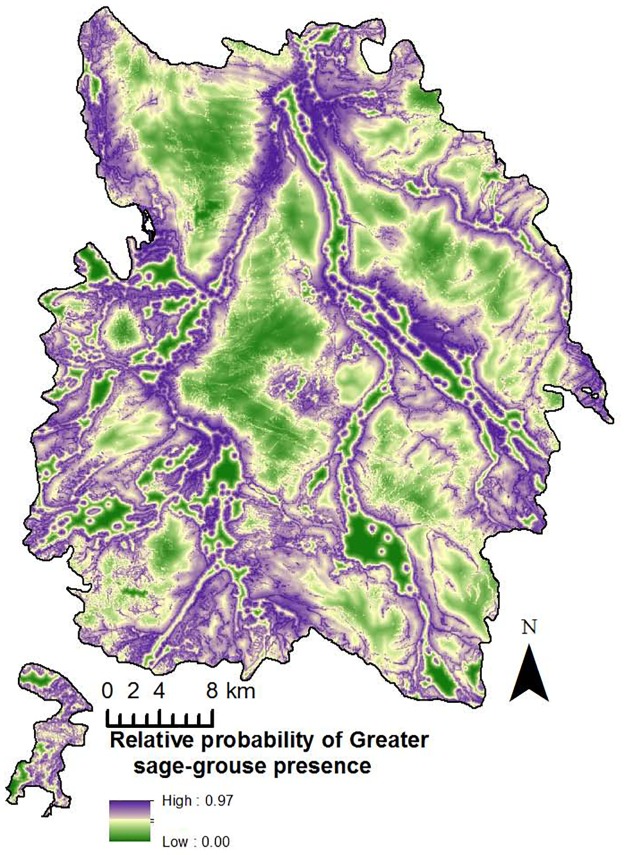
Relative probability of presence during the summer season. Relative probability of female greater sage-grouse presence in North Park, U.S.A. (2010–2011) during the summer season (16 July– 1 September).

Our model-averaged summer model indicates that GRSG strongly selected for grassland cover type, being closer to sagebrush, and higher water density (Table 4). The most important variable was that female GRSG were closer to sagebrush, but had a negative relationship with sagebrush indicating that they were not necessarily located directly within the sagebrush, but along the edges. In addition, female GRSG appeared to be positively influenced by access to water and the edges of irrigated agriculture. The resulting averaged prediction surface illustrates areas along riparian corridors closer to patches of sagebrush were selected by female GRSG during the summer season (Fig 3).

**Table 4:** Coefficients for the top model for the summer season.

	STANDARDIZED COEFFICIENTS				UNSTANDARDIZED COEFFICIENTS				
Variable	β	SE	LCI	UCI	β	SE	LCI	UCI	Odds Ratio
Intercept	- 2.622				- 2.046				
Sagebrush (proportion)	- 0.209	0.080	- 0.365	- 0.053	- 0.517	0.196	- 0.901	- 0.132	0.600
Grassland (proportion)	0.152	0.042	0.070	0.234	1.270	0.348	0.587	1.953	3.562
sagebrush/grassland (proportion)	0.078	0.042	- 0.005	0.160	0.499	0.270	- 0.030	1.027	1.646
distance to sagebrush (km)	- 1.026	0.130	- 1.282	- 0.771	- 6.294	0.800	- 7.861	- 4.727	0.002
distance to agriculture (km)	- 0.468	0.070	- 0.605	- 0.331	- 0.663	0.099	- 0.857	- 0.469	0.515
distance to water (km)	- 0.257	0.059	- 0.373	- 0.142	- 1.186	0.272	- 1.719	- 0.652	0.306
water density (km/km^2^)	0.385	0.056	0.275	0.495	0.354	0.052	0.253	0.455	1.425

Top model standardized and unstandardized coefficients for greater sage-grouse resource selection in North Park, Colorado, U.S.A. (2010–2011) in the summer season (16 July– 1 September), including the standard error (SE), lower (LCI) and upper (UCI) 95% confidence intervals, and odds ratios.

**Fig 4:**
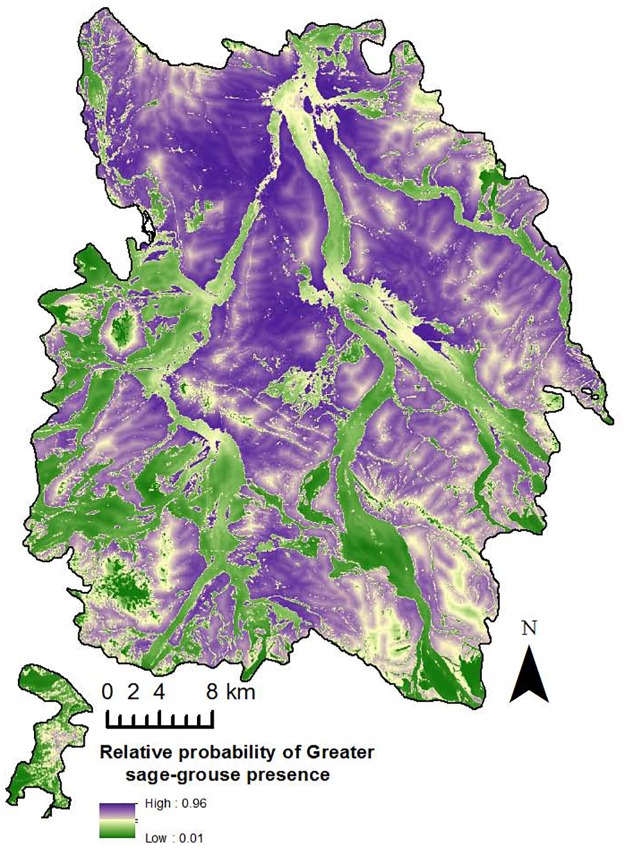
Relative probability of presence during winter season. Relative probability of female greater sage-grouse presence in North Park, U.S.A. (2010–2012) during the winter season (1 October– 1 March).

Our model-averaged winter model includes strong effects of birds selecting sagebrush and sagebrush/grassland landcovers, areas with high water density, and further from irrigated agriculture (Table 5). Smaller effects includes selection for slightly lower elevations, and close to water (Table 5). Sagebrush and sagebrush/grassland were the most important variables for female GRSG in the winter. The averaged prediction surface was very similar to the breeding season prediction surface, although there were more patches of non-habitat within sagebrush dominated landscapes especially in the southern portion of the study area (Fig 4).

**Table 5:** Coefficients for the top model for the winter season.

	STANDARDIZED COEFFICIENTS				UNSTANDARDIZED COEFFICIENTS				
Variable	β	SE	LCI	UCI	β	SE	LCI	UCI	Odds Ratio
Intercept	- 2.202				- 2.038				
Sagebrush (proportion)	1.179	0.062	1.057	1.301	2.967	0.156	2.660	3.273	19.427
Grassland (proportion)	0.087	0.054	- 0.018	0.192	0.816	0.501	- 0.166	1.800	2.262
sagebrush/grassland (proportion)	0.345	0.039	0.270	0.421	2.337	0.262	1.823	2.850	10.035
Elevation (ft)	- 0.433	0.075	- 0.581	- 0.285	- 0.002	0.0003	- 0.002	- 0.001	0.998
distance to agriculture (km)	0.288	0.043	0.203	0.372	0.386	0.058	0.274	0.499	1.472
distance to water (km)	- 0.236	0.04	- 0.320	- 0.151	- 1.111	0.203	- 1.509	- 0.713	0.329
water density (km/km^2^)	0.404	0.051	0.305	0.503	0.382	0.048	0.288	0.475	1.465

Top model standardized and unstandardized coefficients for greater sage-grouse resource selection in North Park, Colorado, U.S.A. (2010–2012) in the winter season (1 October– 1 March) including the standard error (SE), lower (LCI) and upper (UCI) 95% confidence intervals, and odds ratios.

All 3 of our base model-averaged seasonal models validated well based on R^2^ values greater than 0.893 (Table 6). The breeding, winter, and summer models had high average R^2^ values (breeding R^2^ = 0.969; winter R^2^ = 0.928; summer R^2^ = 0.947) indicating good model fit in all seasons. These results emphasize the robustness of the models for all of the seasonal models.

**Table 6:** Model cross validation.

	Breeding		Summer		Winter		Breeding with ED landscape		Breeding with ED local	
	AUC	r_s_	AUC	r_s_	AUC	r_s_	AUC	r_s_	AUC	r_s_
Entire dataset	0.732		0.712		0.754		0.729		0.751	
Validation 1	0.722	0.967	0.692	0.954	0.711	0.893	0.713	0.998	0.735	0.924
Validation 2	0.732	0.964	0.720	0.952	0.741	0.979	0.735	0.985	0.747	0.936
Validation 3	0.728	0.976	0.743	0.952	0.720	0.915	0.719	0.967	0.719	0.851
Validation 4	0.744	0.988	0.684	0.942	0.717	0.948	0.751	0.952	0.797	0.942
Validation 5	0.724	0.952	0.726	0.933	0.706	0.906	0.722	0.994	0.711	0.900
Average	0.730	0.969	0.713	0.947	0.719	0.928	0.728	0.979	0.742	0.911

Cross validated Spearman-rank correlations (r_s_) between resource selection function (RSF) bin ranks and area-adjusted frequencies for individual and average model sets by breeding, summer, and winter seasons and breeding season with energy development (ED) at both the landscape and local scale in North Park, Colorado, U.S.A (2010–2011). Each validation set was based on 20% of the data randomly excluded for model development and then predicting the withheld data. Included are the Area Under the Curve (AUC) estimates for each model set to compare with the original model.

### Energy Development models

For the landscape scale energy development analysis, we ran the model-averaged seasonal models with the addition of uncorrelated energy development variables (distance to oil/gas roads and the energy development index). We found that none of the energy development variables improved fit (based on lower AIC_c_) in the summer or winter season, so we did not continue with producing a prediction surface for these two seasons. We found a small effect using the distance to oil/gas roads variable in the breeding season which was the only energy variable that improved model fit (base breeding model AIC_c_ = 7436, landscape breeding model with energy variables AIC_c_ = 7382, Table 7). The pattern of variable effects was similar to the base breeding model, except for the presence buffers being slightly closer to oil/gas roads than the available buffers (Table 7). The averaged prediction surface from the landscape breeding energy development model indicates very little change from the base breeding model as shown by the similar variable coefficients and the small odds ratio of the oil/gas roads variable (Table 7 and Fig 2B). The model validation R^2^ value for the landscape breeding model including the energy variables performed similarly to the base model (R^2^ = 0.979).

**Table 7:** Coefficients for the top model for the breeding season including energy development variables at the landscape scale*.

	STANDARDIZED COEFFICIENTS				UNSTANDARDIZED COEFFICIENTS				
Variable	β	SE	LCI	UCI	β	SE	LCI	UCI	Odds Ratio
Intercept	- 2.189				- 3.512				
Sagebrush (proportion)	1.272	0.071	1.134	1.411	3.135	0.174	2.794	3.475	22.983
Grassland (proportion)	0.183	0.053	0.078	0.287	1.638	0.477	0.703	2.573	5.144
sagebrush/grassland (proportion)	0.419	0.039	0.343	0.500	2.722	0.253	2.225	3.218	15.210
Elevation (m)	- 0.008	0.035	- 0.160	0.090	- 0.0001	0.0002	- 0.0004	0.002	1.000
distance to agriculture (km)	0.154	0.039	0.078	0.230	0.207	0.052	0.105	0.309	1.230
distance to water (km)	- 0.139	0.037	- 0.211	- 0.067	- 0.644	0.168	- 0.973	- 0.315	0.525
water density (km/km^2^)	0.007	0.024	- 0.093	0.108	0.007	0.048	- 0.088	0.102	1.007
distance to oil roads (km)	- 0.397	0.055	- 0.504	- 0.290	- 0.150	0.021	- 0.191	- 0.110	0.860

Top model standardized and unstandardized coefficients for greater sage-grouse resource selection in North Park, Colorado, U.S.A. (2010–2011) in the breeding season (1 April– 15 July) with additional development variables including the standard error (SE), lower (LCI) and upper (UCI) 95% confidence intervals, and odds ratios.

*Landscape scale analysis includes presence and available buffers across the North Park study area.

For our local scale energy development analysis, we restricted locations (both presence and available) to within 2 km of an active oil well. This clearly reduced the number of locations and we used 455, 333, and 218 breeding, winter, and summer presence locations and 1,700 available locations, respectively. For the summer and winter models, we found that none of the energy development variables improved fit (based on lower AIC_c_). Therefore, we did not generate summer or winter prediction surfaces including the energy development variables.

In contrast, our local breeding season model was improved with the distance to oil/gas roads energy development variable (base breeding model AIC_c_ = 1859, breeding model with energy variables AIC_c_ = 1845; Table 8). This model suggests that female GRSG select areas further from oil/gas roads within the energy development field (within 2 km of energy development). The averaged prediction surface from the local breeding energy development model suggests a loss of breeding habitat especially in the McCallum field area but only in the northeast section of North Park (Fig 2C). The model validation R^2^ value for the breeding model including the energy variables was not as high as the base model (R^2^ = 0.911), but still indicated a robust model.

**Table 8:** Coefficients for the top model for the breeding season including energy development variables at the local scale*.

	STANDARDIZED COEFFICIENTS				UNSTANDARDIZED COEFFICIENTS				
Variable	β	SE	LCI	UCI	β	SE	LCI	UCI	Odds Ratio
Intercept	- 1.638				- 1.357				
Sagebrush (proportion)	1.095	0.141	0.893	1.298	2.784	0.357	2.270	3.298	16.185
Grassland (proportion)	0.250	0.075	0.142	0.358	2.846	0.853	1.617	4.074	17.214
sagebrush/grassland (proportion)	0.707	0.084	0.586	0.828	4.249	0.504	3.524	4.975	70.067
Elevation (ft)	0.207	0.111	0.047	0.368	0.001	0.0008	- 0.0002	- 0.002	1.001
distance to agriculture (km)	- 0.180	0.102	- 0.327	- 0.368	- 0.262	0.149	- 0.476	0.048	0.769
distance to water (km)	- 0.282	0.087	- 0.408	- 0.156	- 1.096	0.341	- 1.587	- 0.605	0.334
water density (km/km^2^)	- 0.500	0.124	- 0.678	- 0.321	- 0.476	0.117	- 0.645	- 0.308	0.621
distance to oil roads (km)	0.347	0.084	0.225	0.468	0.480	0.118	0.311	0.649	1.617

Top model standardized and unstandardized coefficients for greater sage-grouse resource selection in North Park, Colorado, U.S.A. (2010–2011) in the breeding season (1 April– 15 July) with additional development variables including the standard error (SE), lower (LCI) and upper (UCI) 95% confidence intervals, and odds ratios.

* Local scale includes only the presence and available buffers within a 2 km buffer of active well pads.

## Discussion

The GRSG we studied in North Park selected similar habitat variables during the winter and breeding seasons. In both the breeding and winter seasons, sagebrush was the most important variable and in both cases the odds ratio for sagebrush was >19. Another important variable that influenced selection in both seasons was sagebrush/grassland vegetation with odds ratios >10. We found that the averaged relative probability of presence prediction surfaces for breeding and winter seasons were quite similar. Although distance to sagebrush was not considered in either the breeding or winter models due to a correlation with sagebrush, we found that telemetry locations were always within 0.49 km or 0.48 km of sagebrush during the breeding and winter season, respectively (versus up to 1.97 km for available locations). GRSG are sagebrush obligates during both the breeding and winter periods and although lekking occurs in small open areas, nearby sagebrush is critical for escape cover and feeding [33, 54]. Our results highlight the importance of sagebrush and sagebrush/grassland habitats to GRSG in North Park in the breeding and winter seasons and further supports previous landscape scale research (see [5] for review).

Our summer season average prediction surface was different from the other two seasons, mostly because of the slight avoidance of sagebrush. The avoidance we observed is not unexpected based on previous research where sagebrush consists of only a portion of diverse summer habitats [5, 35, 55–58]. Further supporting previous research [3, 35, 55–58], GRSG in North Park used a greater variety of vegetation cover types during summer, but variables associated with water and grassland vegetation types were the most important. Even though non-sagebrush vegetation was important during the summer season, female GRSG were close (< 0.61 km) to sagebrush. As the summer season advances and the understory vegetation begins senescence [56, 58–59], GRSG typically respond by moving to a greater variety of habitats, particularly mesic habitats [5, 27, 35–36, 55–58, 60]. In addition, riparian habitats devoid of woody vegetation provide an abundance of forbs and insects in the summer [27, 33, 54].

Our energy development analysis resulted in conflicting results between the two scales. First, neither the summer nor winter base seasonal models had improved fit by including any of the energy development variables at either scale. Second, distance to oil/gas roads was the only development variable with a slightly significant impact during the breeding season at both scales, but GRSG were closer to roads at a landscape scale, and further from roads at a local scale. Because of the conflicting result, we suggest that overall energy development in North Park may not be at a level that could be significantly impacting female GRSG habitat selection. In the summer and winter seasons, energy development variables had no impact while in the breeding season the impact at both scales was small (odds ratios < 1.5).

Thus, while the breeding season base model was improved with the inclusion of distance to oil/gas roads, we found a positive effect at the landscape scale and a negative effect at a local scale. This resulted in the landscape scale breeding prediction surface showing very little change to the base model whereas the local scale prediction surface showed a reduction in higher relative probability value in energy development areas especially around McCullum Field (Fig 2B). The base breeding model indicates large areas of sagebrush in North Park and many of the oil/gas fields are located within these sagebrush patches (Fig 1). Therefore, one possible explanation for this pattern of use in the landscape breeding energy development model in North Park is that most of the energy development takes place within sagebrush dominated patches relative to the entire study area. The percentage of sagebrush located within 2 km of active well pads is much higher with 65% versus all of the North Park study area (34.1%). This could be indicative that although on a landscape scale in the breeding season birds are closer to oil/gas roads, this could be an artifact of the higher amounts of sagebrush which drive the overall model (sagebrush odds ratio = 23). This pattern of energy development within good sagebrush patches along with a slight avoidance of roads within 2 km of active well pads should cause some concern for wildlife and habitat managers.

Copeland et al. [9] predicted a 7–19% population decline in GRSG across their range from future energy development in sagebrush habitats. Over 68% of the land surface within North Park is sagebrush or sagebrush/grassland mix. North Park has one of three major oil/gas producing basins in Colorado, thus a large portion of North Park has high potential for energy development [61]. For the period of 2008–2027 a range of up to 370 wells could be developed with a possible 1,198 new hectares of disturbance expected [62]. One of the areas considered to have the greatest potential for development and currently in the early stages of exploration is the Niobrara formation in the southern portion of North Park (Fig 1, [62]). One of the limitations to energy development in North Park is the lack of an existing pipeline infrastructure, so if production increases, this could lead to increased development in North Park [62].

Our case study supports the notion that it is critically important for wildlife managers to analyze seasonal habitat use for a species before anthropogenic development occurs. By doing this, we have a better understanding of GRSG habitat selection before intensive energy development. Therefore, we suggest that our initial estimate of seasonal habitat selection for GRSG in North Park at a relatively low energy development level provides a unique way to examine potential impacts of future energy development scenarios. The lack of large effects from our added energy variables also supports using these seasonal resource selection models as baseline data. We suggest that wildlife managers now have an advantage by having our pre-energy (or low density) development seasonal habitat models so they can investigate and monitor future management and conservation scenarios using varying levels of energy development. Our models can help inform future siting of energy development infrastructure to minimize the effects of energy development as well as informing potential mitigation actions [1] throughout the GRSG lifecycle in North Park.

Lastly, our analysis suggests that the scale of analysis can affect the interpretation of the impact of energy development and there have been numerous studies that have found impacts from energy development on GRSG at multiple scales using lek data [6,11,63–65]. The studies document the scale of energy development impact on GRSG from 0.4 km to 20 km [6,11,63–65]. Thus, research designs that specifically focus on management efforts at varying levels of energy development at multiple scales are needed to better understand energy development impacts and the conservation of GRSG.

## Supporting Information

S1 Appendix Vegetation classifications. Basinwide vegetation layer developed by Colorado Division and Wildlife from landsat imagery. Vegetation categories were clipped and grouped specifically for our study area in North Park, Colorado, U.S.A.
(DOCX)

S2 Appendix Top model results. Top models with weights that sum to 95% for the (a) breeding, (b) summer, (c) winter, (d) breeding with energy development at a landscape scale, and (e) breeding with energy development at a local scale for greater sage-grouse in North Park, Colorado, U.S.A. (2010–2012).
(DOCX)

S1 Dataset Seasonal variable values. Values for variables extracted for each seasonal buffer and available buffer used to run the models in this analysis. There are tabs for breeding season, summer season, winter season, and available (7,000 for the breeding and winter season and 6,000 in the summer season were randomly selected). The columns are as follows: bird identifies the individual bird, proportion of irrigated agriculture, proportion of grassland, proportion of sagebrush, proportion of sagebrush/grassland mix, proportion of riparian, elevation (m), distance to water (km), water density (km/km2), distance to sagebrush (km), distance to agriculture (km), distance to oil/gas roads (km), distance to well pads (km), and the energy development index.
(XLSX)

## References

[1] Kirol CP, Beck JL, Huzurbazar SV, Holloran MJ, Miller SN. Identifying greater sage-grouse source and sink habitats for conservation planning in an energy development landscape. Ecol Appl. 2015; 25: 968–990. 26465037 10.1890/13-1152.1

[2] Fedy BC, Doherty KE, Aldridge CL, O’Donnell M, Beck JL, Bedrosian B, et al Habitat prioritization across large landscapes, multiple seasons, and novel areas: An example using greater sage-grouse in Wyoming. Wildlife Monogr. 2014; 190: 1–39.

[3] Schroeder MA, Aldridge CL, Apa AD, Bohne JR, Braun CE, Bunnell SD, et al Distribution of sage-grouse in North America. Condor 2004; 106: 363–376.

[4] U. S. Fish and Wildlife Service. Endangered and threatened wildlife and plants; 12-month finding on a petition to list the greater sage-grouse (Centrocercus urophasianus) as an endangered or threatened; proposed rule: Federal Register, v. 80, pp. 59,857–59,942.

[5] Connelly JW, Rinkes ET, Braun CE. Characteristics of greater sage-grouse habitats: a landscape species at micro- and macro- scales In: Knick ST, Connelly JW, editors.Greater sage-grouse: ecology and conservation of a landscape species and its habitats. Studies in Avian Biology (vol. 38), University of California, Berkeley; 2011 pp. 69–83.

[6] Harju SM, Dzialak MR, Taylor RC, Hayden-Wing LD, Winstead JB. Thresholds and time lags in effects of energy development on greater sage-grouse populations. J Wildl Manage. 2010; 74: 437–448.

[7] Davies KW, Boyd CS, Beck JL, Bates JD, Svejcar TJ, Gregg MA. Saving the sagebrush sea: An ecosystem conservation plan for big sagebrush plant communities. Biol Conserv. 2011;144: 2573–2584.

[8] Bergquist E, Evangelista P, Stohlgren TJ, Alley N. Invasive species and coal bed methane development in the Powder River Basin, Wyoming. Environ Monit Assess. 2007;128: 381–394. 10.1007/s10661-006-9321-7 17016748

[9] Copeland HE, Doherty KE, Naugle DE, Pocewicz A, Kiesecker JM. Mapping Oil and Gas Development Potential in the US Intermountain West and Estimating Impacts to Species. 2009; PLoS ONE 4: e7400 10.1371/journal.pone.0007400 19826472 PMC2756592

[10] Naugle DE, Doherty KE, Walker BL, Holloran MJ, Copeland HE. Energy development and greater sage-grouse In: Knick ST, Connelly JW, editors. Greater sage-grouse: ecology and conservation of a landscape species and its habitats. Studies in Avian Biology (vol. 38), University of California, Berkeley; 2011 pp. 489–503.

[11] Walker BL, Naugle DE, Doherty KE. Greater sage-grouse population response to energy development and habitat loss. J Wildl Manage. 2007; 71:2644–2654.

[12] Holloran MJ, Fedy BC, Dahlke J. Winter habitat use of greater sage-grouse relative to activity levels at natural gas well pads. J Wildl Manage. 2015; 79:630–640.

[13] Lyon AG, Anderson SH. Potential gas development impacts on sage-grouse nest initiation and movement. Wildl Soc Bull. 2003; 31:486–491.

[14] Doherty KE, Naugle DE, Walker BL, Graham JM. Greater sage-grouse winter habitat selection and energy development. J Wildl Manage. 2008; 72:187–195.

[15] Smith KT, Kirol CP, Beck JL, Blomquist FC. Prioritizing winter habitat quality for greater sage-grouse in a landscape influenced by energy development. Ecosphere. 2014; 5:1–20.

[16] Colorado Greater Sage-Grouse Steering Committee. Colorado greater sage-grouse conservation plan. Denver (CO): Colorado Division of Wildlife; 2008.

[17] Colorado Oil and Gas Conservation Commission (COGCC) [Internet] Denver (CO): Well surface location data: wells. c2014 –[cited 2012 Sept 1]. Available from http://cogcc.state.co.us/data2.html#/downloads.

[18] Braun CE, Oedekoven OO, Aldridge CL. Oil and gas development in western North America: effects on sagebrush steppe avifauna with particular emphasis on sage-grouse. Trans North Am Wildl Nat Resour Conf. 2002; 67:337–349.

[19] Walker BL, Apa AD, Eichhoff K. Mapping and prioritizing seasonal habitats for greater sage-grouse in Northwestern Colorado. J Wildl Manage. 2016; 80:63–67.

[20] Dzialak MR, Olson CV, Harju SM, Webb SL, Winstead JB. Temporal and hierarchical spatial components of animal occurrence: conserving seasonal habitat for greater sage-grouse. Ecosphere 2012; 3:1–17.

[21] U.S. Census Bureau [Internet] Washington D.C.:State and county quickfacts: Jackson County, CO. c2010—[cited 2015 Aug 31]. Available from: http://quickfacts.census.gov.

[22] Giesen KM, Schoenberg TJ, Braun CE. Methods for trapping sage-grouse in Colorado. Wildl Soc Bull. 1982; 10:224–231.

[23] Wakkinen WL, Reese KP, Connelly JW, Fischer RA. An improved spotlighting technique for capturing sage-grouse. Wildl Soc Bull. 1992; 20:425–426.

[24] Stokland JN, Halvorsen R, Stoa B. Species distribution modeling- Effect of design and sample size of pseudo-absence observations. Ecol Modell. 2011; 222:1800–1809.

[25] Northrup JM, Hooten MB, Anderson CR Jr, Wittemyer G. Practical guidance on characterizing availability in resource selection functions under a use-availability design. Ecol. 2013; 94: 1456–1463. 10.1890/12-1688.1 23951705

[26] Aldridge CL, Boyce MS. Linking occurrence and fitness to persistence: habitat based approach for endangered greater sage-grouse. Ecol Appl. 2007; 17:508–526 17489256 10.1890/05-1871

[27] Doherty KE, Naugle DE, Walker BL. Greater sage-grouse nesting habitat: the importance of managing at multiple scales. J Wildl Manage. 2010; 74:1544–1553.

[28] Rice MB, Apa TD, Phillips ML, Gammonly JH, Petch BF, Eichhoff K. Analysis of regional species models based on radio-telemetry datasets from multiple small-scale studies. J Wildl Manage. 2013; 77:821–831.

[29] Colorado Division of Water Resources[Internet] Denver, CO: Colorado’s Decision Support Systems, Division 6, District 47 irrigated lands. c2015—[cited 2015 Sept 14]. Available from http://cdss.state.co.us/GIS/Pages/Division6YampaWhite.aspx.

[30] Blomberg EJ, Sedinger JS, Atamian MT, Nonne DV. Characteristics of climate and landscape disturbance influence the dynamics of greater sage-grouse populations. Ecosphere. 2012; 3:1–20.

[31] Tedrow L, Weber KT. NDVI changes over a calendar year in the rangelands of southeast Idaho. In: Weber KT, Davis K, editors. Final report: assessing post-fire recovery of sagebrush-steppe rangelands in Southweastern Idaho (NNX08AO90G), pp 105–116.

[32] Sveum CM, Crawford JA, Edge DW. Use and selection of brood rearing habitat by sage-grouse in south-central Washington. Great Basin Nat. 1998; 58:344–351.

[33] Connelly JW, Schroeder MA, Sands AR, Braun CE. Guidelines to manage sage-grouse populations and their habitats. Wildl Soc Bull. 2000; 28:967–985.

[34] Atamian MT, Sedinger JS, Heaton JS, Blomberg EJ. Landscape-level assessment of brood rearing habitat for greater sage-grouse in Nevada. J Wildl Manage. 2010; 74:1533–1543.

[35] Dunn PO, Braun CE. Summer habitat use by adult and juvenile sage-grouse. J Wildl Manage. 1986; 50: 228–235.

[36] Klott JH, Lindzey FG. Brood habitats of sympatric sage-grouse and Columbian sharp tailed grouse in Wyoming. J Wildl Manage. 1990; 54:84–88.

[37] R Core Team. R: a language and environment for statistical computing. Version 2.15[software]. 2013. Available from: http://www.R-project.org/.

[38] Gillies CS, Hebblewhite M, Nielsen SE, Krawchuk MA, Aldridge CL, Frair JL, et al Application of random effects to the study of resource selection by animals. J Anim Ecol. 2006; 75: 887–898. 17009752 10.1111/j.1365-2656.2006.01106.x

[39] McAlpine CA, Rhodes JR, Bowen ME, Lunney D, Callaghan JG, Mitchell DL, et al Can multiscale models of species’ distribution be generalized from region to region? A case study of koala. J Appl Ecol. 2008; 45:558–567.

[40] Burnham KP, Anderson DR. Model selection and multimodel inference: a practical information-theoretic approach. New York: Springer-Verlag; 2002.

[41] Manly BFJ, McDonald LL, Thomas DL, McDonald TL, Erickson WP. Resource selection by animals: statistical design and analysis for field studies. Dordrecht, The Netherlands: Kluwer Academic Publishers; 2002.

[42] Schielzeth H. Simple means to improve the interpretability of regression coefficients. Methods Ecol Evol. 2010; 1: 103–113.

[43] Anderson DR. Model based inference in the life sciences: a primer on evidence. New York:Springer Science+Business Media, LLC; 2008.

[44] Aldridge CL, Saher DJ, Childers TM, Stahlnecker KE, Bowen ZH. Crucial nesting habitat for Gunnison sage-grouse: A spatially explicity hierarchical approach. J Wildl Manage. 2012; 76:391–406.

[45] Colorado Department of Transportation [Internet]. Denver (CO): Online Transportation information system data catalog: local and major roads. [cited 2012 Sept 1]. Available from: http://dtdapps.coloradodot.info/otis/catalog.

[46] Remington TE, Braun CE. How surface coal mining affects sage-grouse, North Park, Colorado In: Emerick JC, editor. Proceedings, Issues and Technology in the Management of Impacted Western Wildlife. Thorne Ecological Institute 1991; 5:128–132.

[47] Manier DJ, Wood DJA, Bowen ZH, Donovan RM, Holloran MJ, Juliusson LM, et al. Summary of science, activities, programs, and policies that influence the rangewide conservation of greater sage-grouse (Centrocercus urophasianus). Reston (VA):U.S. Geological Survey Open-File Report; 2013. Report No: 2013–1098. Available from http://pubs.usgs.gov/of/2013/1098/.

[48] Boyce MS, Vernier PR, Nielsen SE, Schmiegelow FKA. Evaluating resource selection functions. Ecol Modell. 2002; 157:281–300.

[49] Johnson CJ, Seipand DR, Boyce MS. A quantitative approach to conservation planning: using resource selection functions to map the distribution of mountain caribou at multiple spatial scales. J Appl Ecol. 2004; 41:238–251.

[50] Hirzel AH, LeLay G, Helfer V, Randin C, Guisan A. Evaluating the ability of habitat suitability models to predict species presences. Ecol Modell. 2006; 199: 142–152.

[51] Johnson CJ, Niesen SE, Merrill EH, McDonald TL, Boyce MS. Resource selection functions based on use-availability data: theoretical motivation and evaluation methods. J Wildl Manage. 2006; 70:347–357.

[52] Johnson CJ, Gillingham MP. Sensitivity of species-distribution models to error, bias, and model design: an application to resource selection functions for woodland caribou. Ecol Modell. 2008; 213:143–155.

[53] Wiens TS, Dale BC, Boyce MS, Kershaw GP. Three way k-fold cross validation of resource selection functions. Ecol Modell. 2008; 244–255.

[54] Connelly JW, Knick ST, Braun CE, Baker WL, Beever EA, Christiansen T, et al Conservation of Greater Sage-Grouse: a synthesis of current trends and future management In: Knick ST, Connelly JW, editors. Greater sage-grouse: ecology and conservation of a landscape species and its habitats. Studies in Avian Biology (vol. 38), University of California, Berkeley; 2011 p. 549–563.

[55] Patterson RL. The sage-grouse in Wyoming. Denver (CO): Sage Books, Inc.; 1952.

[56] Klebenow DA, Gray GM. Food habitat of juvenile sage-grouse. J Range Manage. 1968; 21:80–83.

[57] Drut MS, Pyle WH, Crawford JA. Diets and food selection of sage-grouse chicks in Oregon. J Range Manage. 1994; 47:90–93.

[58] Fischer RA, Reese KP, Connelly JW. Influence of vegetal moisture content and nest fate on timing of female sage-grouse migration. Condor. 1996; 98: 868–872.

[59] Savage DE. Relation of sage-grouse to upland meadow in Nevada. Completion report. Reno (NV): Nevada Division of Wildlife; 1969. Report No.:Project W-39-R-9, Job 12.

[60] Hausleitner D. Population dynamics, habitat use and movements of greater sage-grouse in Moffat county, Colorado[Thesis]. Moscow (ID): University of Idaho; 2003.

[61] Bureau of Land Management (BLM) Northwest Colorado greater sage-grouse approved resource management plan amendment. Lakewood (CO): Department of the Interior Bureau of Land Management Colorado State Office;2015.

[62] Bureau of Land Management (BLM) (2009) Reasonable foreseeable development 2008–2027 oil and gas activities in the kremmling field office. Kremmling (CO); 2009.

[63] Hollaran MJ. Greater sage-grouse (Centrocercus urophasianus) population response to natural gas field development in western Wyoming. University of Wyoming [Dissertation]. Larimie (WY): University of Wyoming; 2005.

[64] Tack JD. Sage-grouse and the human footprint: implications for conservation of small and declining populations. [Thesis]. Missoula (MT): University of Montana; 2009.

[65] Taylor RL, Tack JD, Naugle DE, Mills LS. Combined effects of energy development and disease on greater sage-grouse. 2013; PLoS ONE 8(8): e71256 10.1371/journal.pone.0071256 23940732 PMC3734021

