# Supplementary material for: Seasonal Habitat Use by Greater Sage-Grouse (Centrocercus urophasianus) on a Landscape with Low Density Oil and Gas Development
Source: PLoS One. 2016 Oct 27;11(10):e0165399. doi: 10.1371/journal.pone.0165399 (PMC5082953; doi:10.1371/journal.pone.0165399)
Supplement: S1 Appendix — Basinwide vegetation layer developed by Colorado Division and Wildlife from landsat imagery. Vegetation categories were clipped and grouped specifically for our study area in North Park, Colorado, U.S.A. (DOCX) [file pone.0165399.s001.docx]

**S1 Appendix.** **Vegetation classifications.** Basinwide vegetation layer developed by Colorado Division and Wildlife from landsat imagery. Vegetation categories were clipped and grouped specifically for our study area in North Park, Colorado, U.S.A.

**Basinwide category class used for North Park models**

High density residential areas, lawns, planted trees. residential

Irrigated crops and fields. irrigated agriculture

Rangeland dominated by annual and perennial grasses. grassland

Rangeland codominated by grasses and forbs. grassland

Disturbed or overgrazed rangeland. grassland

Sparsely vegetated grasslands, 10-40% vegetation. bare

Sagebrush(*Artemisia spp.)* with rabbitbrush(*Chrysothammus spp*.), bitterbrush (*Purshia spp.)* sagebrush

Low elevation shrubland dominated by greasewood(*Sarcobatus vermiculatus*). greasewood

Shrubland dominated by bitterbrush. bitterbrush

Codominate sagebrush shrubland and perennial grassland. sagebrush/grassland

Codominate sagebrush/mesic mtn shrub mixed with grass/forb. sagebrush

Deciduous forest dominated by aspen (*Populus tremuloides*). aspen

Codominate aspen and gambel oak (*Quercus gambelii*) deciduous woodland. aspen

Coniferous forest dominated by douglas fir (*Pseudotsuga menziesii*). forest

Coniferous forest dominated by lodgepole pine (*Pinus contorta*). forest

Coniferous forest dominated by limber pine (*Pinus flexilis*). forest

Coniferous forest co-dominated by lodgepole pine,

engleman spruce (*Picea engelmannii*), and white fir (*Abies concolor*) forest

Mixed forest codominated by aspen and lodgepole pine. forest

Talus and scree slopes, nearly 100% rock. talus

Bare soil and fallow agriculture fields. bare

High elevation meadows co-dominated by grass and forbs alpine

Shrub riparian areas consisting primarily of shrub willows. riparian

Shrub riparian areas dominated by shrub willow species. riparian

Non-woody riparian areas consisting primarily of sedges (*Carex spp*.). herbaceous riparian

Lakes, reservoirs, rivers, streams. Water
