# Supplementary material for: Seasonal Habitat Use by Greater Sage-Grouse (Centrocercus urophasianus) on a Landscape with Low Density Oil and Gas Development
Source: PLoS One. 2016 Oct 27;11(10):e0165399. doi: 10.1371/journal.pone.0165399 (PMC5082953; doi:10.1371/journal.pone.0165399)
Supplement: S2 Appendix — Top models with weights that sum to 95% for the (a) breeding, (b) summer, (c) winter, (d) breeding with energy development at a landscape scale, and (e) breeding with energy development at a local scale for greater sage-grouse in North Park, Colorado, U.S.A. (2010–2012). (DOCX) [file pone.0165399.s002.docx]

**S2 Appendix**: **Top model results.** Top models with weights that sum to 95% for the (a) breeding, (b) summer, (c) winter, (d) breeding with energy development at a landscape scale, and (e) breeding with energy development at a local scale for greater sage-grouse in North Park, Colorado, U.S.A. (2010-2012).

**a).** Model selection results in the 95% model averaged weight set using all subsets of model parameters in for the breeding season models for greater sage-grouse in North Park, Colorado, U.S.A. (2010-2011): sagebrush (sb), distance to agriculture (agdt), elevation (e), grass (g), sagebrush/grassland (sbgr), water density (wtds), and distance to water (wtdt).

| **Models** | **K** | **Log likelihood** | **AIC_c_** | **∆AIC_c_** | **weight** |
| --- | --- | --- | --- | --- | --- |
| agdt+e+g+sb+sbgr+wtdt | 8 | - 3710.21 | 7436.42 | 0.00 | 0.44 |
| agdt+e+g+sb+sbgr+wtds+wtdt | 9 | - 3709.97 | 7437.93 | 1.52 | 0.21 |
| agdt+g+sb+sbgr+wtdt | 7 | - 3712.04 | 7438.08 | 1.66 | 0.19 |
| agdt+g+sb+sbgr+wtds+wtdt | 8 | - 3712.04 | 7440.08 | 3.66 | 0.07 |
| agdt+e+g+sb+sbgr | 7 | - 3713.35 | 7440.70 | 4.28 | 0.05 |
| e+g+sb+sbgr+wtds+wtdt | 8 | - 3713.14 | 7442.27 | 5.85 | 0.02 |
| agdt+e+g+sb+sbgr+wtds | 8 | - 3713.33 | 7442.66 | 6.24 | 0.02 |

**b).** Model selection results in the 95% model averaged weight set using all subsets of model parameters in for the summer season models for greater sage-grouse in North Park, Colorado, U.S.A. (2010-2011): sagebrush (sb), distance to agriculture (agdt), distance to sagebrush (sbdt), grass (g), sagebrush/grassland (sbgr), water density (wtds), and distance to water (wtdt).

| **Models** | **K** | **Log likelihood** | **AIC_c_** | **∆AIC_c_** | **weight** |
| --- | --- | --- | --- | --- | --- |
| agdt+g+sb+sbgr+sbdt+wtds+wtdt | 9 | -2630.46 | 5278.93 | 0.00 | 0.5891 |
| agdt+g+sb+sbdt+wtds+wtdt | 8 | -2631.92 | 5279.85 | 0.92 | 0.3711 |

**c).** Model selection results in the 95% model averaged weight set using all subsets of model parameters in for the winter season models for greater sage-grouse in North Park, Colorado, U.S.A. (2010-2011): sagebrush (sb), distance to agriculture (agdt), distance to elevation(e), grass (g), sagebrush/grassland (sbgr), water density (wtds), and distance to water (wtdt).

| **Models** | **K** | **Log likelihood** | **AIC_c_** | **∆AIC_c_** | **weight** |
| --- | --- | --- | --- | --- | --- |
| agdt+e+g+sb+sbgr+wtds+wtdt | 9 | -3730.08 | 7478.16 | 0.00 | 0.56 |
| agdt+e+sb+sbgr+wtds+wtdt | 8 | -3731.33 | 7478.65 | 0.49 | 0.44 |

**d).** Model selection results in the 95% model averaged weight set using all subsets of model parameters in for the breeding season including energy development at the landscape scale variables models for greater sage-grouse in North Park, Colorado, U.S.A. (2010-2011): sagebrush (sb), distance to agriculture (agdt), elevation (e), grass (g), sagebrush/grassland (sbgr), water density (wtds), distance to water (wtdt), and distance to energy development roads (dvrddt).

| **Models** | **K** | **Log likelihood** | **AIC_c_** | **∆AIC_c_** | **weight** |
| --- | --- | --- | --- | --- | --- |
| agdt+g+dvrrddt+sb+sbgr+wtdt | 8 | -3683.16 | 7382.32 | 0.00 | 0.56 |
| agdt+elev+g+dvrrddt+sb+sbgr+wtdt | 9 | -3683.00 | 7384.01 | 1.69 | 0.24 |
| agdt+g+dvrrddt+sb+sbgr+wtdt+wtdt | 9 | -3683.15 | 7384.30 | 1.98 | 0.21 |

**e).** Model selection results in the 95% model averaged weight set using all subsets of model parameters in for the breeding season including energy development variables at the local scale models for greater sage-grouse in North Park, Colorado, U.S.A. (2010-2011): sagebrush (sb), distance to agriculture (agdt), elevation (e), grass (g), sagebrush/grassland (sbgr), water density (wtds), distance to water (wtdt), energy development density (dvds), and distance to energy development roads (dvrddt).

| **Models** | **K** | **Log likelihood** | **AIC_c_** | **∆AIC_c_** | **weight** |
| --- | --- | --- | --- | --- | --- |
| agdt+e+g+dvrddt+sb+sbgr+wtds+wtdt | 10 | -912.42 | 1844.84 | 0.00 | 0.27 |
| agdt+e+g+dvds+dvrddt+sb+sbgr+wtds+wtdt | 11 | -911.89 | 1845.79 | 0.94 | 0.17 |
| e+g+dvrddt+sb+sbgr+wtds+wtdt | 9 | -913.94 | 1845.87 | 1.03 | 0.16 |
| agdt+g+dvrddt+sb+sbgr+wtds+wtdt | 9 | -914.10 | 1846.20 | 1.35 | 0.14 |
| e+g+dvds+dvrddt+sb+sbgr+wtds+wtdt | 10 | -913.16 | 1846.32 | 1.48 | 0.13 |
| agdt+g+dvds+dvrddt+sb+sbgr+wtds+wtdt | 10 | -913.62 | 1847.25 | 2.41 | 0.08 |
| g+dvrddt+sb+sbgr+wtds+wtdt | 8 | -915.96 | 1847.92 | 3.08 | 0.06 |
